# Intradural-Extramedullary Solitary Fibrous Tumor/Hemangiopericytoma with a Negative Result on Fluorodeoxyglucose-Positron Emission Tomography/Computed Tomography

**DOI:** 10.1155/2019/3926903

**Published:** 2019-11-22

**Authors:** Yusuke Tomomatsu, Yoichi Iizuka, Tokue Mieda, Junko Hirato, Hiromi Koshi, Hiroyuki Sonoda, Sho Ishiwata, Yohei Kakuta, Akira Honda, Tsuyoshi Tajika, Hirotaka Chikuda

**Affiliations:** ^1^Department of Orthopaedic Surgery, Gunma University Graduate School of Medicine, 3-39-22, Showa, Maebashi, Gunma 371-8511, Japan; ^2^Clinical Department of Pathology, Gunma University Hospital, 3-39-22, Showa, Maebashi, Gunma 371-8511, Japan

## Abstract

Intradural-extramedullary solitary fibrous tumor/hemangiopericytoma (SFT/HPC) is a rare entity. SFT/HPCs can recur after surgery, even if a benign histology of the tumor is observed. We herein report a 68-year-old woman with intradural-extramedullary SFT/HPC. On magnetic resonance imaging (MRI), the intradural-extramedullary mass was isointense on T1-weighted images and hypointense on T2-weighted images with heterogeneous gadolinium enhancement. Whole-body fluorodeoxyglucose-positron emission tomography/computed tomography (^18^F-FDG-PET/CT) was also performed, showing no accumulation. We performed surgery for the intradural-extramedullary mass, and the pathological findings of the resected specimen were a benign histology consistent with World Health Organization (WHO) grade I SFT/HPC. She had no evidence of tumor recurrence three years after the surgery for intradural-extramedullary SFT/HPC. ^18^F-FDG-PET/CT before surgery may be useful for predicting the postoperative behavior of spinal SFT/HPCs.

## 1. Introduction

Solitary fibrous tumors are rare mesenchymal tumors, first described in 1931 by Klemperer and Coleman as a primary neoplasm arising from the pleura [1]. Hemangiopericytoma, which derives from the pericytes around capillaries and postcapillary venules, was first described by Stout and Murray in 1942 [2]. Although both solitary fibrous tumor and hemangiopericytoma can arise in any soft tissues of the human body, including the spinal cord, the World Health Organization Classification of Tumors of the Central Nervous System (CNS WHO) assigned the combined term solitary fibrous tumor/hemangiopericytoma (SFT/HPC) to such lesions in 2016 based on the fact that both tumors share inversions at 12q13, leading to STAT6 nuclear expression [3]. SFT/HPCs can recur after surgery, even if the tumors have a benign histology consistent with grade I SFT/HPC.

We herein report a case of intradural-extramedullary SFT/HPC at the level of the thoracic spine, which had a negative result on fluorodeoxyglucose-positron emission tomography/computed tomography (^18^F-FDG-PET/CT) before surgery.

## 2. Case Report

**Figure 1:**
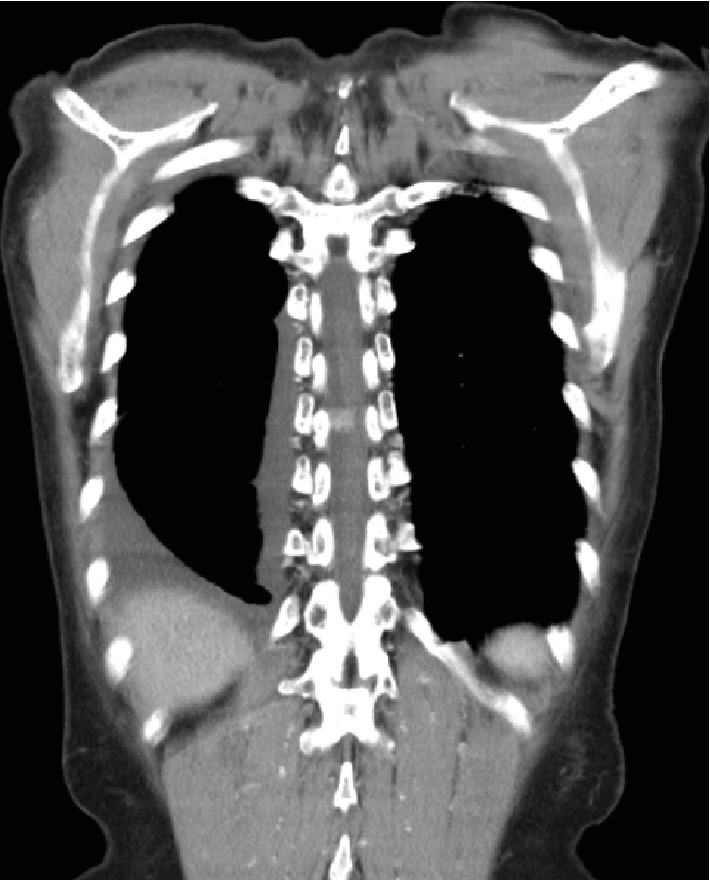
Follow-up CT with contrast medium one month after surgery for clear cell renal cell carcinoma showing a homogeneously enhanced mass without intralesional calcifications.

A 68-year-old woman underwent lobectomy for lung adenocarcinoma and cryosurgery for clear cell renal cell carcinoma 4 months after the surgery for the lung cancer. Follow-up CT imaging with contrast medium one month after the surgery for the kidney cancer detected a homogenously enhanced mass without intralesional calcifications in the spinal canal at the level of T9 (Figure 1), although she had no symptoms related to the mass lesion.

**Figure 2:**
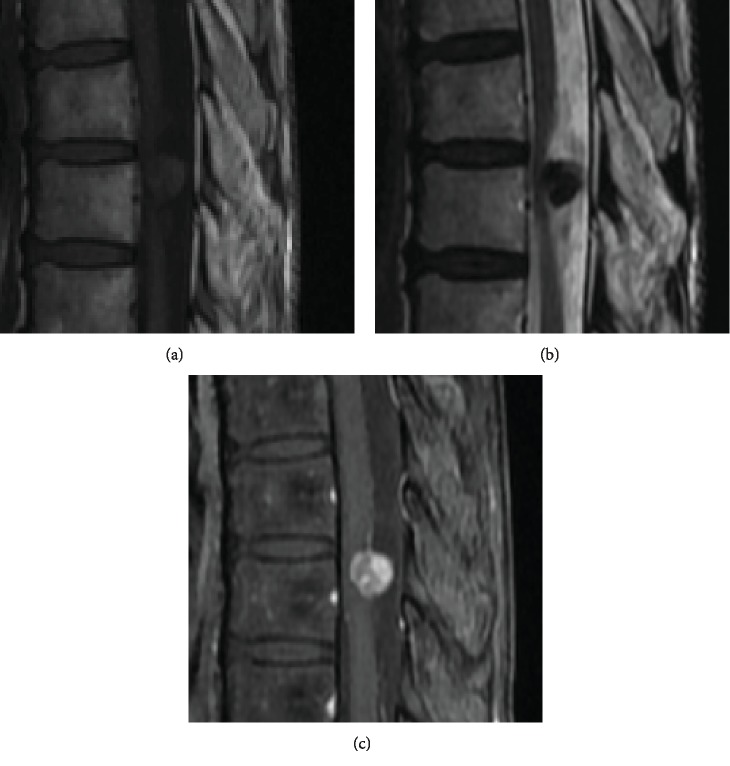
MRI findings showing an intradural-extramedullary mass with isointensity on T1-weighted images (a) and hypointensity on T2-weighted images (b), enhanced heterogeneously with gadolinium (c).

Additional magnetic resonance imaging (MRI) revealed an intradural-extramedullary mass with isointense changes on T1-weighted images and hypointense changes on T2-weighted images as well as heterogeneous gadolinium enhancement (Figure 2).

**Figure 3:**
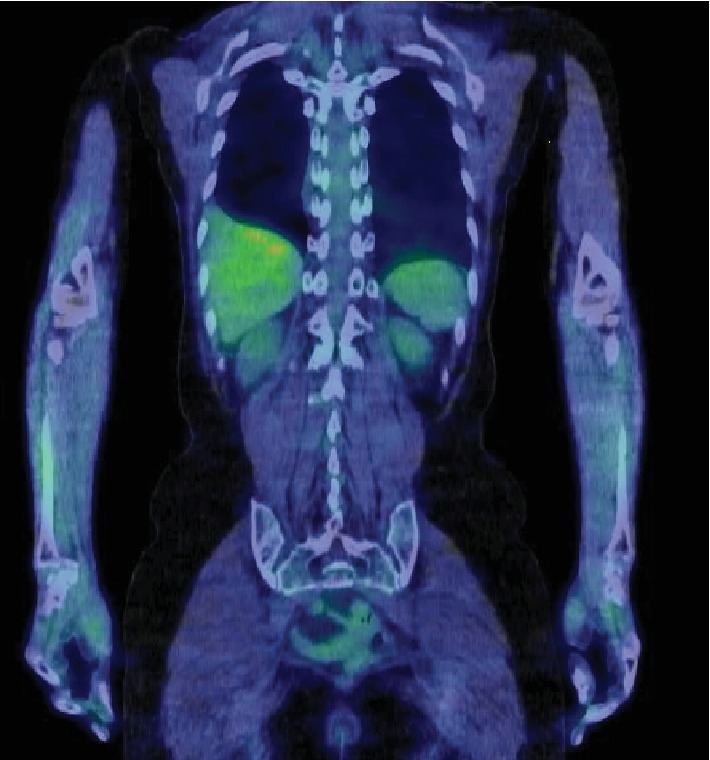
^18^F-FDG-PET/CT findings showing no accumulation in the intradural-extramedullary site at the level of T9.

Whole-body ^18^F-FDG-PET/CT was performed to differentiate between benign primary spinal cord tumor and spinal cord metastasis associated with lung or kidney cancer, and there was no accumulation on ^18^F-FDG-PET/CT (Figure 3).

**Figure 4:**
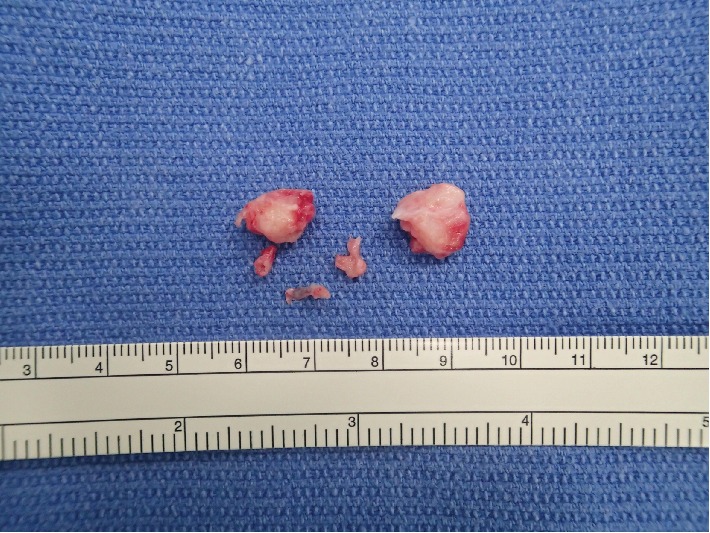
White-yellowish hard tumor was resected in a piecemeal fashion.

She developed back pain and numbness in the lower limbs two months after the kidney surgery. A neurological examination revealed hyperreflexia in the muscle stretch reflex in the lower limbs despite normal muscle strength and no bladder dysfunction. Based on the ^18^F-FDG-PET/CT findings indicating no spinal cord metastasis, we performed surgery for the intradural-extramedullary spinal cord tumor at the level of T9. First, we performed laminectomies of T8 and T9 and a biopsy of a white-yellowish hard tumor located in the intradural-extramedullary site for intraoperative pathological consultation (Figure 4), which showed no evidence of malignancy.

**Figure 5:**
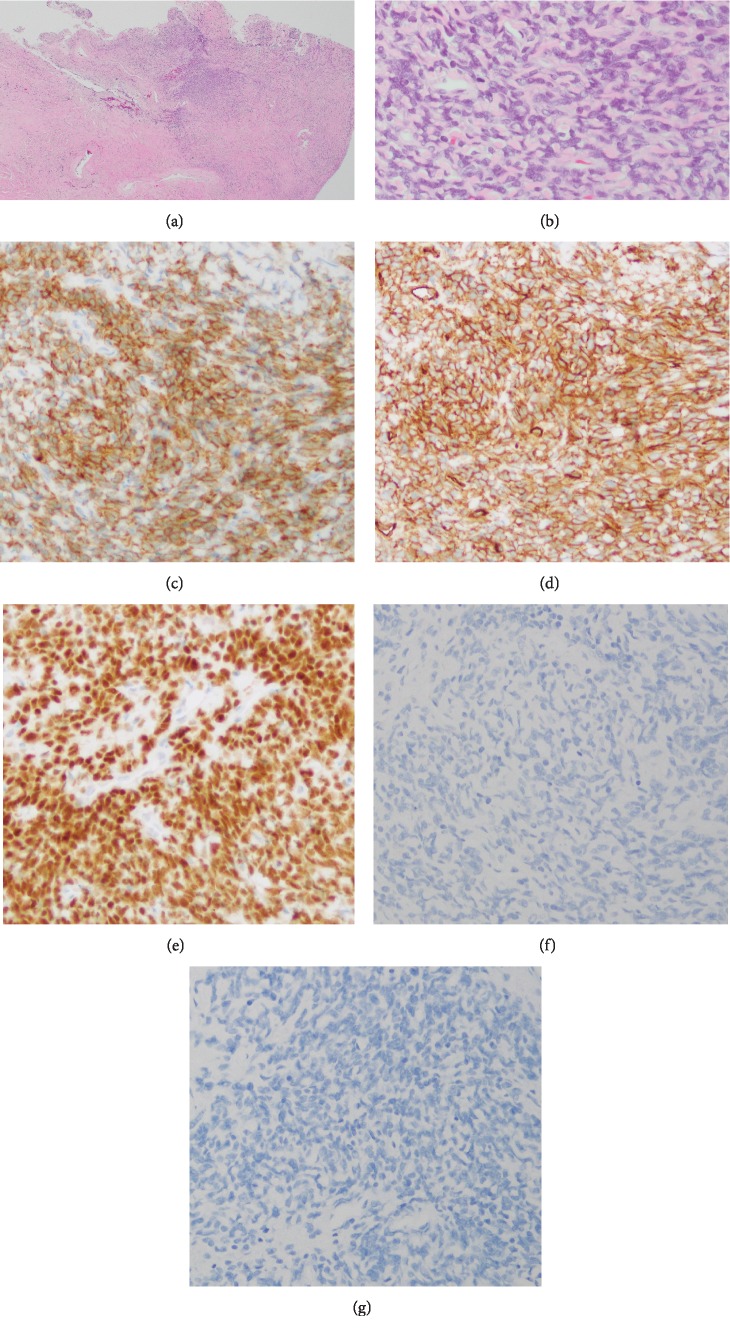
The tumor consisted of spindle cells with ovoid to spindle-shaped nuclei, arranged without a pattern with stromal hyalinized collagen (HE staining) (a, b). On immunohistochemical staining, CD34 (c), bcl-2 (d), and STAT6 (e) were positive, while S-100 protein (f) and EMA (g) were negative.

Finally, we achieved gross total resection of the tumor using a microscope despite the fact that the resection was done in a piecemeal fashion because of the adhesion between the spinal cord and the spinal cord tumor. A histological examination of the resected specimen revealed that the tumor showed a patternless architecture with alternating hypocellular and hypercellular areas and deposition of hyalinized collagen fiber. The tumor was composed of spindle cells with ovoid to spindle-shaped nuclei. Mitosis and necrosis were generally scarce. On an immunohistochemical examination, CD34, bcl-2, and STAT6 were positive, while S-100 protein, neurofilament protein, and EMA were negative (Figure 5). Those pathological findings were consistent with grade I SFT/HPC.

**Figure 6:**
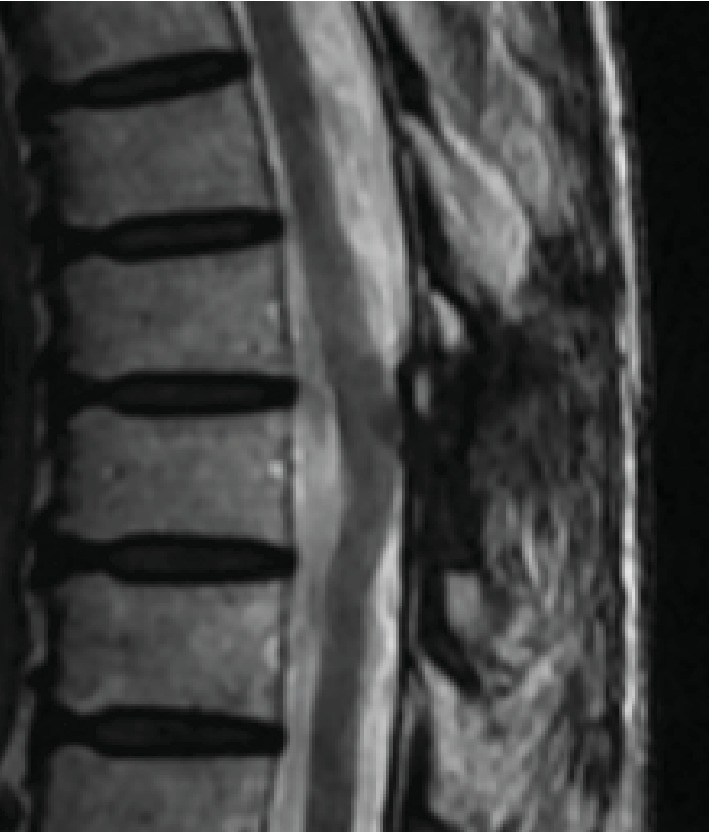
MRI of the thoracic spine showed no evidence of tumor recurrence three years after the surgery for intradural-extramedullary SFT/HPC.

Although she still complained of slight back pain three years after the surgery, she had no neurological deficits in the lower limbs. MRI of the thoracic spine showed no evidence of tumor recurrence (Figure 6), and enhanced whole-body CT also showed no evidence of metastasis or recurrence of SFT/HPC and other tumors.

## 3. Discussion

Spinal SFT/HPC is a rare entity, and only about 90 cases have been reported to date. The majority of spinal SFT/HPC cases have occurred in adults with a slight male predominance. Spinal SFT/HPCs most often occur in the thoracic region followed by the cervical and lumbar regions, and most cases occur at intradural-extramedullary sites [4–6]. Although spinal SFT/HPCs are treated with surgery in the overwhelming majority of cases and gross total resection can be curative [7], Fargen et al. reported that 14.8% of spinal SFT/HPC cases recurred after gross total resection [8].

Regarding the radiological diagnosis of spinal SFT/HPCs, it is often difficult to differentiate intradural-extramedullary SFT/HPCs from other intradural-extramedullary spinal cord tumors preoperatively because of a lack of specific radiological findings for SFT/HPCs [4, 5].

At MRI, SFT/HPCs are usually isointense on both T1- and T2-weighted images and enhanced with contrast because they are vascular tumors [9]. The MRI findings in the present case showed that the tumor was isointense on T1-weighted images and hypointense in T2-weighted images and enhanced heterogeneously with contrast. The MRI pattern was therefore different from the typical findings of the two most common intradural-extradural tumors, schwannoma and meningioma, in which the lesions are usually hypo- or isointense on T1-weighted images and hyperintense on T2-weighted images, with both enhanced with contrast [10]. Therefore, we considered another spinal lesion besides schwannoma or meningioma in this case, preoperatively. However, we did not suspect SFT/HPC because of its rarity, although the MRI findings more closely resembled the findings typical of SFT/HPC than those of schwannoma or meningioma.

To our knowledge, this is the first report to describe the ^18^F-FDG-PET/CT findings of spinal SFT/HPCs, although there have been several reports regarding the ^18^F-FDG-PET/CT findings of benign and malignant SFT/HPCs in the pleura, liver, extremities, and kidney [11–15]. Those associated reports indicated that benign SFT/HPC lesions had no or only slight accumulation, whereas malignant SFT/HPCs had a high accumulation on ^18^F-FDG-PET/CT, although there was a case report of a false-negative malignant SFT/HPC in an extremity [7]. In the present case, grade I SFT/HPC with a good postoperative course showed no accumulation on ^18^F-FDG-PET/CT, suggesting that this imaging modality may be useful for the preoperative prediction of the postoperative behavior of spinal SFT/HPCs.

## 4. Conclusion

We performed surgery for intradural-extramedullary SFT/HPC with a preoperative negative result on ^18^F-FDG-PET/CT. The patient had no evidence of tumor recurrence three years after the surgery.
